# Integrated Care: A Person-Centered and Population Health Strategy for the COVID-19 Pandemic Recovery and Beyond

**DOI:** 10.5334/ijic.7536

**Published:** 2023-12-11

**Authors:** Ayodele Odutayo, Kevin Smith, Carolyn Gosse, Melissa Chang, Shiran Isaacksz, Christopher T. Chan

**Affiliations:** 1 Division of Nephrology, University Health Network and Department of Medicine, University of Toronto, Toronto, Ontario, Canada; 2 Connected Care, University Health Network, Toronto, Ontario, Canada

**Keywords:** integrated care, COVID-19, hospital re-admission

## Abstract

The COVID-19 pandemic has mandated a re-imagination of how healthcare is administered and delivered, with a view towards focusing on person-centred care and advancing population health while increasing capacity, access and equity in the healthcare system. These goals can be achieved through healthcare integration. In 2019, the University Health Network (UHN), a consortium of four quaternary care hospitals in Ontario, Canada, established the first stage of a pilot program to increase healthcare integration at the institutional level and vertically with other primary, secondary and tertiary institutions in the Ontario healthcare system. Implementation of the program was accelerated during the COVID-19 pandemic and demonstrated how healthcare integration improves person-centred care and population health; therefore serving as the foundation for a health system response for the COVID-19 pandemic recovery and beyond.

## Introduction

Since the outset of the COVID-19 pandemic, healthcare systems have faced unprecedented disruptions to the delivery of clinical care. Due to the transmissibility and virulence of the SARS-CoV-2 virus, the COVID-19 pandemic simultaneously increased demands on primary, secondary and tertiary care. This includes increasing demands on acute and critical care as well as magnifying racial and socioeconomic inequities in health [1].

The multidimensional impact of the COVID-19 pandemic on the healthcare system mandates a re-imagination of how healthcare is administered and delivered, with a view towards focusing on person-centred care and advancing population health while increasing capacity, access, and equity. In response to the challenges of the COVID-19 pandemic, the University Health Network (UHN)—the largest quaternary university hospital in Ontario, Canada – established a healthcare integration program which achieves the dual purpose of person-centred care while improving population health; thereby producing a sustainable health system response to the COVID-19 pandemic and preparing the healthcare system for the future.

## Health Care Integration at the University Health Network as a Strategic Person-centred Response to Covid-19

The UHN is a quaternary care centre based in Toronto, Ontario that provides surgical and medical care to highly complex patients. UHN is made up of four hospitals with unique clinical specialization. These include the Toronto General Hospital (specializing in multi-organ transplant), Toronto Western Hospital (Neurosurgery and Neurology), Princess Margaret Hospital (Oncology) and Toronto Rehab Institute (Rehabilitation Medicine). The UHN has 1286 patient beds and from 2021–2022 provided care for 108,569 emergency visits. Clinical care at UHN is provided by a mix of dedicated clinically focused physicians and allied health team members, as well as academic physicians who also have a research appointment at the University of Toronto. Patients move seamlessly through all sites of UHN through the use of a unified electronic health record system.

Prior to the COVID-19 pandemic in 2019, the UHN established the first stage of a pilot program to increase healthcare integration at the institutional level. Implementation of the program was accelerated during the pandemic, in large part due to resource constraints on the existing health system infrastructure at UHN. The pandemic created an urgent need for innovative person-centred solutions that optimized patient care and increased clinical capacity. Prior barriers such as inertia related to overhauling administration, funding and pathways for clinical care could be deconstructed as part of the transition to virtual care. Indeed, the initial pilot of the UHN transitional care program for COVID-19 focused on virtual care clinics, transitional care programs as well as population level programs for the distribution or prophylactic antibody treatments for COVID-19 in a decentralized manner. As a case study, an integrated care program was introduced for the General Internal Medicine service from January to February 2022. The program provided an integrated care pathway that enrolled patients with COVID-19 pneumonia, CHF or COPD with mild – moderate symptoms. Patient who were nearing discharge could be discharged home earlier and be supported by an integrated team of providers including homecare supports (e.g. nursing, personal support workers) and care coordination support, for up to 2-weeks post-discharge. This integrated care team provided the care needed to support each patients full recovery, with the potential to refine the level of homecare support required by patients. Of 36 people enrolled in the UHN pilot integrated care program, only 3 of 36 (8%) required re-admission to hospital.

The success of this program lies in addressing a key point of transition from the hospital to the home, wherein patients and families consistently face challenges in identifying how to connect with their hospital-based team. Prior to the integrated care program, healthcare institutions were solely responsible for submitting the request for homecare and patients and families played the role of care coordination. This resulted in gaps in care and ultimately an avoidable re-admission to hospital. Table 1 uses the Kaiser Permanente Pyramid of Care as well as the Health Quality Ontario Hospital to Home Quality Standard to demonstrate how the healthcare integration program at UHN bridges an important gap between the hospital and discharge home, thereby achieving the dual goals of person-centred care while improving population health.

**Table 1:** Case Study of the University Health Network Health Integration Program.


PATIENT ACUITY	SERVICE PROVISION	UHN SPECIFIC PROGRAMS DURING THE COVID-19 PANDEMIC

Highly Complex Patients	Case Management	Information-Sharing on Admission Bi-directional and timely information sharing amongst primary care and home and community care providers, relevant specialists with admitting team. Transitions of Care: A named health care professional is responsible for timely transition planning, coordination, and communication. Written transition plan, developed by and agreed upon in partnership with patients and circle of care shared within 48 hrs of discharge. Circle of care involved in planning and developing a written transition plan. Follow-up medical care with their primary care provider and/or a medical specialist is coordinated and booked before leaving. People with no primary care provider are provided with assistance to find one. Patient, Family, and Caregiver Education, Training, and Support: Information and support provided to manage their health care needs; education and training offered to manage at home, including guidance on community-based resources, medications, and medical equipment. Assess for the type, amount, and appropriate timing of home care and community support services are needed (including caregivers.) When needed, services are arranged before leaving and in place upon return home.

High Risk Patient	Acute Illness Management	Services oriented around information sharing on admission, transitions of care and patient, family and caregiver education, training and support Eligibility for General Internal Medicine Integrated Care Pathway Assessment and suitability for management at home Discharged home with 3 consecutive nursing visits post-discharge Ongoing monitoring with as needed visits for up to 2 weeks

Stable Chronic Disease	Disease Management	COVID-19 Connected Virtual Care Clinic Nurse led virtual care clinic for assessment and managing symptoms of COVID-19 for patients with stable chronic disease (e.g. transplant recipients) Distribution of pulse oximeters to facilitate home monitoring

Population Wide Prevention	Promotion and Prevention	Coordination of Primary and Secondary Prevention Programs Centralized prescription of monoclonal antibodies to prevent progressive to severe SARS-CoV-2 pneumonia Creation of vaccine clinics in line with Provincial mandates


This table leverages the Kaiser Permanente Pyramid of Care as well as the Health Quality Ontario Hospital to Home Quality Standard to demonstrate how health care integration at the University Health network achieves the dual goals of person-centred care while improving population health.

Given the noted benefits of the pilot integrated care program, the program has been rapidly expanded into a broader integrated care initiative that involves other clinical divisions at UHN as the emergency phase of COVID-19 pandemic has ended. This includes dedicated integrated care programs in transplant medicine, surgery and oncology. To meet the resource requirements of this expansion, the integrated care team is now made up of a dedicated program director, project manager, integrated care leads, homecare team and transitional care nurse practitioners. All patients in the integrated care program have access to an integrated care phone line where they can speak to a clinician 24 hours a day. The principal offering of the transitional care program includes providing virtual out of hospital follow up via patient led self-monitoring or clinician led biometric monitoring and homecare supports such as at home nursing and physiotherapy. Importantly, the transitional care program also adopted a risk based approach, where services offered to patients are determined based on their clinical acuity and risk for re-admission.

To ensure the sustainability of the integrated care program, collaborations have been established with other providers including an expanded partnership with the provincially mandated organization for coordinating community health in Ontario Canada. Similarly, to provide advanced care in the home, UHN will continue to collaborate with all stakeholders including community paramedicine team, virtual pharmacy, multimedia patient education program where our integrated care team will monitor patients in the transitional care program in their homes and community to ensure the best possible clinical outcomes with the most efficient hospital stay. Ultimately, these efforts to find alignment within a broader regional effort for health system integration and to manage resource offerings based on patients’ acuity have enabled the transitional care program to outlast the COVID-19 pandemic.

## Future Directions

Going forward, the program at UHN will be leveraged to create new clinical and research infrastructure that can provide comprehensive and real time data capture to inform the continued quality and improvement of the healthcare integration program. Risk prediction models and artificial intelligence models can be developed using this data and incorporated into integrated care programs to better identify the needs of patients and families. These insights will also for allow for dedicated efforts to examine racial and socioeconomic inequities within the healthcare system and how these inequities are propagated through transitions in care. Integrated care programs may therefore be employed as strategies to close important racial and socioeconomic gaps that contribute to poor health outcomes as evidenced by the COVID-19 pandemic. Finally, the program can also be leveraged to create research infrastructure for interventional studies by identifying patients interested in supporting research and providing a platform to rapidly scale up these studies, both in the context of usual clinical practice and for a future pandemic.

## Conclusion

The present COVID 19 pandemic has demonstrated the ineffectiveness of siloed healthcare. The pivotal steps that enabled UHN to introduce an integrated care program included leveraging virtual care as an initial cost-effective strategy to demonstrate the improvement in health system capacity with a transitional care program and aligning the integrated care imitative with broader regional health system priorities. Given the ongoing increase in healthcare demands coupled with finite resources in personnel and infrastructure, this scalable model for healthcare system integration may benefit other jurisdictions.
